# SARS‐CoV‐2 Is Linked to Brain Volume Loss in Multiple Sclerosis

**DOI:** 10.1002/acn3.70091

**Published:** 2025-05-29

**Authors:** Tomas Uher, Dominika Stastna, Ingrid Menkyova, Vaclav Capek, Jiri Lindner, Petra Nytrova, Jan Krasensky, Eliza Varju, Miguel D'haeseleer, Eva Kubala Havrdova, Dana Horakova, Manuela Vaneckova, Niels Bergsland

**Affiliations:** ^1^ Department of Neurology and Centre of Clinical Neuroscience, First Faculty of Medicine Charles University in Prague and General University Hospital Prague Czechia; ^2^ Department of Neurology, Faculty of Medicine Slovak Medical University Bratislava Slovakia; ^3^ Department of Radiology, First Faculty of Medicine Charles University in Prague and General University Hospital Prague Czechia; ^4^ Danish Multiple Sclerosis Registry, Department of Neurology Copenhagen University Hospital‐Rigshospitalet Glostrup Denmark; ^5^ Universitair Ziekenhuis Brussel (UZ Brussel), Department of Neurology Brussels Belgium; ^6^ National Multiple Sclerose Centrum (NMSC) Melsbroek Belgium; ^7^ Vrije Universiteit Brussel (VUB), Center for Neurosciences (C4N), neuroprotection and Neuromodulation (NEUR) Research Group Brussels Belgium; ^8^ Buffalo Neuroimaging Analysis Center, Department of Neurology, Jacobs School of Medicine and Biomedical Sciences University at Buffalo, State University of New York Buffalo New York USA; ^9^ IRCCS, Fondazione Don Carlo Gnocchi ONLUS Milan Italy

**Keywords:** brain atrophy, brain lesion, COVID‐19, MRI, multiple sclerosis, SARS‐CoV‐2

## Abstract

**Objective:**

The impact of SARS‐CoV‐2 infection on brain and spinal cord pathology in patients with multiple sclerosis (pwMS) remains unclear. We aimed to describe changes in brain lesion activity and brain and spinal cord volumes following SARS‐CoV‐2 infection.

**Methods:**

We included 177 pwMS (570 MRI scans) diagnosed with and tested positive for SARS‐CoV‐2 infection between August 2020 and May 2021. All patients were free of clinical disease activity, disease‐modifying therapy changes, and corticosteroids during the study. MRI scans were performed using a standardized protocol on a 3‐Tesla scanner. We analyzed the effect of SARS‐CoV‐2 on brain lesion load accrual and brain and spinal cord volume measures using adjusted mixed‐effect models.

**Results:**

During SARS‐CoV‐2 infection, patients had a median disease duration of 14.2 years, a median age of 44.9 years, and a median Expanded Disability Status Scale of 2.0. SARS‐CoV‐2 infection did not lead to any changes in the number or volume of T1 or T2 lesions in the brain. However, SARS‐CoV‐2 was associated with an increased whole brain (*B* = −0.17; SE = 0.08; *p* = 0.028), grey matter (*B* = −0.25; SE = 0.12; *p* = 0.040), and cortical grey matter volume loss (*B* = −0.32; SE = 0.13; *p* = 0.014). Greater ventricular enlargement following SARS‐CoV‐2 infection was evident only in individuals over the age of 40 (interaction of age vs. ventricular enlargement: *B* = 0.17; SE = 0.05; *p* = 0.0003). Only patients with more severe SARS‐CoV‐2 infection showed a reduction in mean upper cervical cord area (MUCCA) (*B* = 1.14; SE = 0.52; *p* = 0.030).

**Interpretation:**

SARS‐CoV‐2 infection in clinically stable pwMS was linked to increased neuronal tissue loss.

## 1 Introduction

Recent research showed that infection with severe acute respiratory syndrome coronavirus 2 (SARS‐CoV‐2) may be associated with accelerated clinical disease activity in patients with multiple sclerosis (pwMS) [1, 2, 3]. However, little is known about the effect of SARS‐CoV‐2 on radiological markers of disease activity in pwMS [4]. It is known that SARS‐CoV‐2 may lead to structural and functional alterations in the brain tissue of COVID‐19 patients [5, 6, 7]. In healthy individuals, even mild infections were associated with mild cognitive decline and brain structural changes, including reduction of whole brain volume, enlargement of brain ventricles, and reduction of grey matter thickness, especially in the brain regions that are functionally connected to the primary olfactory cortex, such as the orbitofrontal cortex and the parahippocampal gyrus [8].

Multiple sclerosis (MS) is a chronic autoimmune and neurodegenerative disease of the central nervous system (CNS) [9, 10]. Accumulation of lesion burden, but also accelerated brain and spinal cord atrophy, is an important correlate of disease progression. Therefore, abnormal magnetic resonance imaging (MRI) findings are used to monitor disease activity in clinical practice [11, 12]. The accumulation of focal lesions and accelerated brain and spinal cord volume loss can occur not only due to MS activity but also as a result of physiological changes [13, 14] or comorbidities such as arterial hypertension, ischemic injury, migraine, and others [15, 16, 17, 18]. Therefore, the identification of factors other than MS responsible for CNS damage is of high importance in improving the interpretation of MRI findings in patients with MS (pwMS).

A recent study investigated the effect of SARS‐CoV‐2 on the brain in 14 pwMS. Although this study did not show an acceleration of whole brain or total grey matter volume loss or increased lesion activity following SARS‐CoV‐2 infection, likely due to the small sample size and low statistical power, it did reveal regional volume loss in the parahippocampal gyri [4]. This finding is consistent with previous studies on the general population [8]. To our knowledge, there are no other studies investigating the impact of SARS‐CoV‐2 on the MRI of pwMS. In our previous research, we demonstrated increased clinical activity in patients following SARS‐CoV‐2 infection within our cohort from a large MS centre [3]. Building on these findings, this study investigates the impact of SARS‐CoV‐2 on brain and spinal cord MRI in a clinically stable sample of pwMS from the same MS centre. The goal is to uncover any underlying changes that may not be immediately apparent through clinical observation alone.

## 2 Materials and Methods

### 2.1 Study Population

**FIGURE 1:**
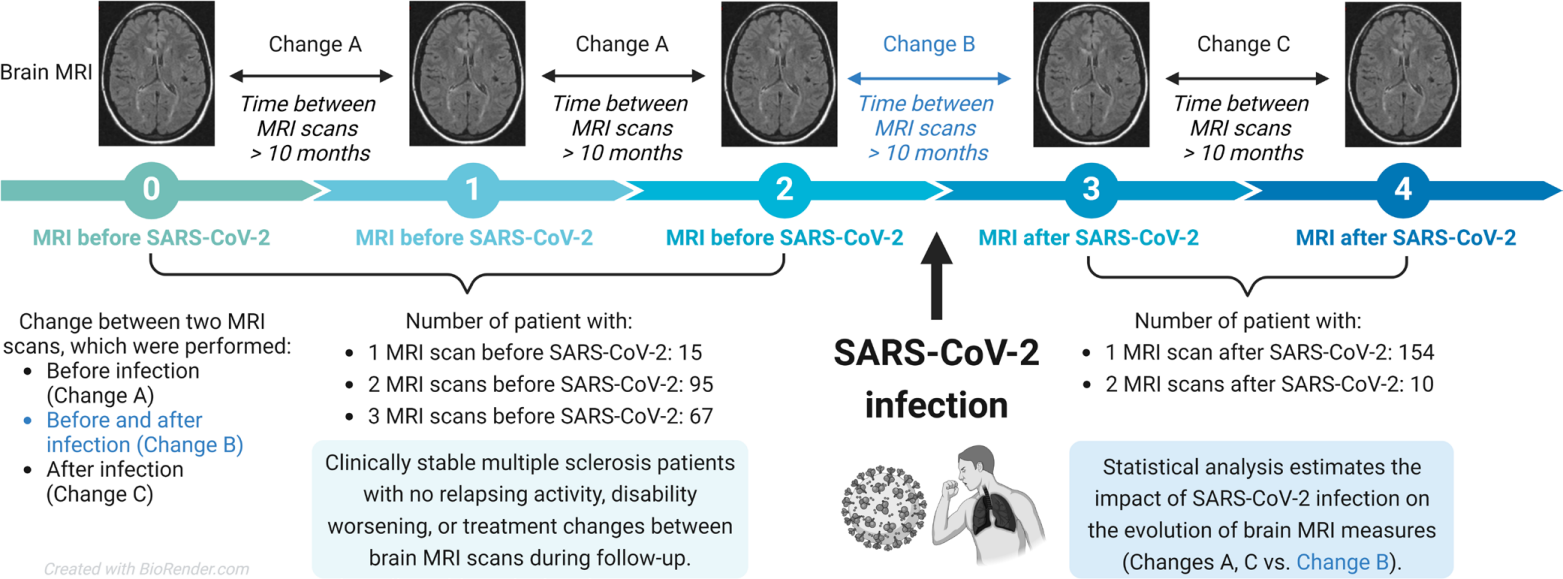
Study design.

In this single‐centre retrospective observational study, we included pwMS diagnosed with and tested positive for SARS‐CoV‐2 infection between August 2020 and May 2021 at the MS Center of the General University Hospital in Prague. The methodology of data collection has already been described in previous publications [3, 19, 20]. In fact, in 4 (2.2%) patients, only polymerase chain reaction (PCR) positivity for COVID‐19 was present without any signs or symptoms of SARS‐CoV‐2 infection. The diagnosis of MS was made according to McDonald's 2017 criteria. Although a spinal tap may not be required for the diagnosis of MS in patients with clinical or imaging evidence meeting the dissemination in time criterion, all patients included in the study underwent cerebrospinal fluid analysis at the time of diagnosis to exclude alternative diagnoses [21]. We included adults (18 years of age or more) who had clinically stable disease. Clinical stability was defined as the absence of both clinical relapses and disability worsening between the first and the last MRI scan during the follow‐up. Disability worsening was defined as any increase in Expanded Disability Status Scale [EDSS] between the first and the last MRI scan during the follow‐up (EDSS change ≤ 0). All patients included in the study had only one documented episode of SARS‐CoV‐2 infection, confirmed by PCR testing. In addition, all patients had no changes in their disease‐modifying treatments (DMT) and had not been administered high‐dose corticosteroids between the first and the last MRI scan during the follow‐up. Only a single patient initiated a new DMT < 6 months before the first MRI scan of the study. Additionally, no patient received high‐dose corticosteroids < 4 weeks before the first MRI scan. Initially, we examined 177 stable pwMS with a history of SARS‐CoV‐2 and brain MRI before and after SARS‐CoV‐2 (Figure 1). All patients underwent regular quarterly or half‐yearly clinical visits with an EDSS evaluation.

Clinical and demographic data in this study from our MS center were collected via the Czech Republic nationwide registry (ReMuS). The guarantor of expertise of ReMuS is the Section for Neuroimmunology and Liquorology of the Czech Neurological Society. Data in ReMuS are collected using the standardized software iMed. Before the release of the coded patient‐level data to investigators, there is a multi‐level quality control process. ReMuS is based on informed consent; thus, it is possible to use retrospective data for scientific and research purposes without requiring new approvals [22]. The study was carried out according to the Declaration of Helsinki, and all patients provided their written informed consent.

### 2.2 Acquisition and Analysis of MRI

Brain MRI scans were performed using a standardized protocol on a 3‐Tesla scanner (MAGNETOM Skyra; Siemens Healthcare, Erlangen, Germany) with a standard 20‐channel head coil. The protocol included 3D T2‐weighted fluid‐attenuated inversion recovery (FLAIR‐SPACE [TR = 5000 ms, TE = 397 ms, TI = 1800 ms, resolution = 1.0 mm iso, TA = 3:17]), along with 3D T1‐weighted magnetization‐prepared acquisition gradient echo (MPRAGE [TR = 2300 ms, TE = 2.26 ms, TI = 732 ms, FA = 8°, resolution = 1.0 mm iso, TA = 5:30]) sequences [23].

MRI examinations were performed for routine monitoring of disease activity. The time between two consecutive scans was at least 10 months (Figure 1), and MRI scans were performed at least 1 month after the diagnosis of SARS‐CoV‐2 infection.

MRI images were preprocessed with N4 (part of the Advanced Normalisation Tools (ANTs)) and aligned using the FMRIB linear image registration tool (FLIRT; http://www.fmrib.ox.ac.uk/fsl/fslwiki/FLIRT). The 3D‐T1 images were then inpainted using the FSL Lesion Filling Tool (https://fsl.fmrib.ox.ac.uk/fsl/fslwiki/lesion_filling). The percent changes in the volume of the whole brain and ventricles were calculated using SIENA and VIENA, respectively, while cortical, grey, and white matter volume changes were obtained using the SIENAX‐MTP method [24]. Thalamic volumes were estimated using FMRIB's Integrated Registration and Segmentation Tool (FIRST) [25]. The number and volume of T2‐FLAIR and T1 hypointensity (i.e., ‘black hole’ [BH]) lesions were obtained using an in‐house deep learning classification algorithm [26]. All lesion masks were quality‐reviewed and manually corrected as necessary. The spinal cord volume was quantified by measuring the mean upper cervical cord area (MUCCA) using a semi‐automatic edge‐finding tool implemented in ScanView.cz, as described previously [27].

### 2.3 Statistical Analysis

Analyses were performed using R statistical software, version 4.3.1 (http://www.R‐project.org). The normality of the distribution was assessed using the Shapiro–Wilk test and visual inspection of histograms.

All patients contributed with two or more timepoints (Figure 1 and Table 1); therefore, we used linear mixed models that allowed us to analyse repeated measures data (lme4 package, version 1.1–34, largest package, version 3.1–3). We investigated the difference in MRI measures before and after SARS‐CoV‐2 (treated as categorical independent variable). For each MRI measure, we calculated the relative change (for regional and whole brain volume and MUCCA) or the absolute change (for lesion numbers and volumes) between the two timepoints. These MRI changes were then annualised based on the number of years between the two timepoints, and these annualised MRI changes were included in the statistical models (treated as continuous dependent variables). A random intercept was specified for each subject, and the models were adjusted for time from SARS‐CoV‐2, sex, age, EDSS, DMT status at the time of infection, severity of the infection, and use of anti‐SARS‐CoV‐2 treatment (all treated as independent variables). In the models, we also included interactions between SARS‐CoV‐2 and all independent variables, except for the severity of the infection and the use of antiviral treatment. The DMT status was considered as a categorical variable (1: untreated; 2: low‐efficacy [dimethyl fumarate, glatiramer acetate, interferon β, teriflunomide]; 3: moderately‐high efficacy [cladribine, fingolimod]; and 4: high efficacy monoclonal antibodies [anti‐CD20, alemtuzumab, and natalizumab]). Details about the structure of the mixed models are shown in Table S1. Due to the exploratory character of the study, *p*‐values < 0.05 were considered statistically significant and were not reported with correction for the false discovery rate.

**TABLE 1:** Characteristics of patients with multiple sclerosis at the visit before SARS‐CoV‐2.

Variable	Median (IQR)
Number of patients	177
Demographics at SARS‐CoV‐2	
Females (*n*; %)	134 (75.7%)
Age (years)	44.9 (39.5; 51.6)
Disease duration (years)	14.2 (9.9; 19.8)
Clinical status at SARS‐Cov‐2	
EDSS	2.0 (1.5; 3.5)
Relapsing–remitting phenotype	143 (80.8%)
Secondary progressive phenotype	34 (19.2%)
Number of relapses during last 12 months	0 (0; 0)
Disease‐modifying treatment	
Injectables (*n*; %)	71 (40.1%)
Orals (*n*; %)	65 (36.7%)
High‐efficacy monoclonal antibodies (*n*; %)	28 (15.8%)
No disease‐modifying treatment (*n*; %)	13 (7.3%)
MRI	
Number of MRI scans	570
Number of longitudinal MRI scans	393
Number of MRI scans per patient	3.2
Number of patients with two MRI scans	28
Number of patients with three MRI scans	82
Number of patients with four MRI scans	67
Number of patients with MRI activity during last 12 months	0 (0; 0)
Number of T1 lesions at scan before SARS‐Cov‐2	6.0 (6.0; 13.0)
Number of T2 lesions at scan before SARS‐Cov‐2	16.0 (16.0; 23.0)
MUCCA (cm^2^) at scan before SARS‐Cov‐2 (*n* = 158)	0.83 (0.75; 0.90)
SARS‐CoV‐2 severity^a^	
Asymptomatic	6 (3.4%)
Symptomatic without suspected pneumonia	146 (82.5%)
Suspected pneumonia defined by both dry cough and shortness of breath	13 (7.3%)
Radiologically confirmed pneumonia (chest X‐ray or CT scan)	8 (4.5%)
Need of supplemental oxygen	2 (1.1%)
Need of non‐invasive ventilation or high‐flow oxygen therapy	2 (1.1%)
Anti‐SARS‐CoV‐2 treatment	
Number of patients receiving antiviral therapy	10 (5.6%)
Convalescent plasma	3 (1.7%)
Casirivimab/imdevimab (REGEN‐COV)	2 (1.1%)
Bamlanivimab	3 (1.7%)
Remdesivir	2 (1.1%)
Patients vaccinated against SARS‐CoV‐2	0 (0; 0)

*Note:* Unless otherwise indicated, numbers are reported as median and interquartile ranges.

Abbreviations: EDSS, expanded disability status scale; MUCCA, mean upper cervical cord area; *n*, number; SARS‐CoV‐2, severe acute respiratory syndrome coronavirus 2.

^a^ Worst‐case scenario applies.

## 3 Results

### 3.1 Patient Characteristics

We included 177 patients with 570 brain MRI scans. Of these, 135 patients had at least two MRI scans before and one scan after SARS‐CoV‐2. The mean time between two consecutive scans was 411 days (median 372; IQR [360; 413]; minimum 311 and maximum 819 days). The median duration of the disease was 14.2 years, the median age was 44.9 years, and the median EDSS score was 2.0. All patients were free of clinical disease activity during the study. Most of the patients (92.6% of timepoints) were treated with DMT. Only two (1.1%) patients infected with SARS‐CoV‐2 required supplemental oxygen. A detailed description of the patient cohort is provided in Table 1.

### 3.2 Changes in Lesion Burden Following SARS‐CoV‐2

We did not find associations between SARS‐CoV2 and the evolution of the number or volume of T1 and T2 lesions (Table 2 and Table S2).

**TABLE 2:** Impact of the SARS‐CoV‐2on MRI measures in clinically stable patients with multiple sclerosis.

MRI measure		Statistical analysis		
		*B*	SE	*p*
Lesions (annualised)	T1 lesion number change	0.02	0.39	0.97
	T1 lesion volume change (mm^3^)	−100.92	51.46	0.051
	T2 lesion number change	1.02	0.78	0.19
	T2 lesion volume change (mm^3^)	−27.85	57.96	0.63
Brain volumes (annualised)	Ventricles volume change (%)	−7.11	2.17	0.0013
	Whole brain volume change (%)	−0.17	0.08	0.028
	Grey matter volume change (%)	−0.25	0.12	0.040
	Cortical volume change (%)	−0.32	0.13	0.014
	White matter volume change (%)	0.57	0.71	0.42
	Thalamic volume change (%)	0.49	1.47	0.74
Spinal cord (annualised)	MUCCA change (%)	1.14	0.52	0.030

*Note:* The structure of the statistical models is shown in Table S1.

Abbreviations: B, unstandardised beta coefficient from the linear mixed model; MUCCA, mean upper cervical cord area; SE, standard error.

### 3.3 Changes in Brain and Spinal Cord Volume Following SARS‐CoV‐2

**FIGURE 2:**
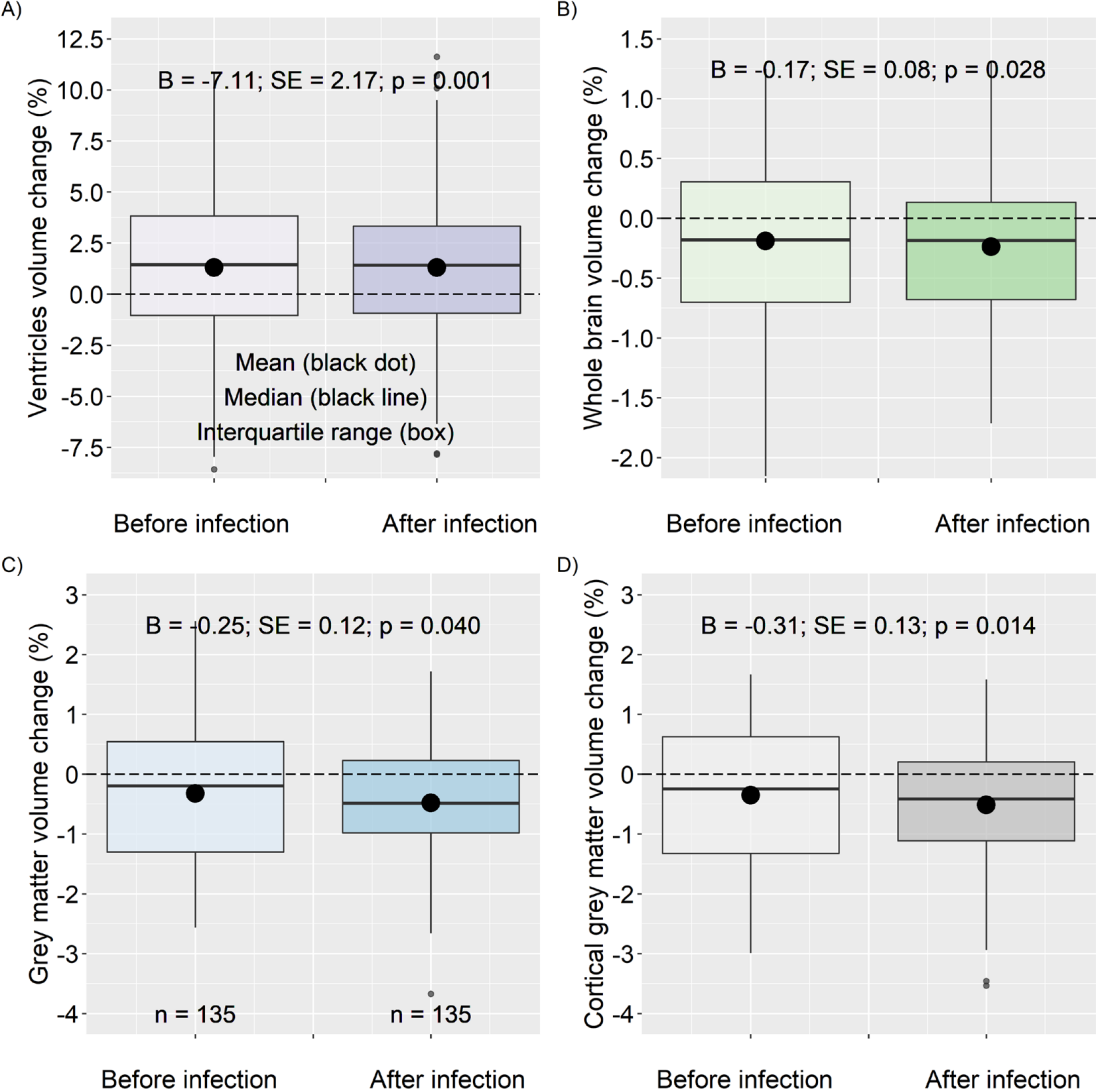
Changes in brain volume measures before and after SARS‐CoV‐2. Show results only for the 135 patients with at least two scans available before and one after infection. Some outliers are not shown.

SARS‐CoV‐2 was associated with an increased whole brain (unstandardised beta coefficient from the adjusted linear mixed models [*B*] = −0.17; SE = 0.08; *p* = 0.028), grey matter (*B* = −0.25; SE = 0.12; *p* = 0.040), and cortical grey matter volume loss (*B* = −0.32; SE = 0.13; *p* = 0.014) (Table 2, Figure 2). SARS‐CoV‐2 was not associated with volume changes in the white matter or thalamus (Table 2, Table S2).

The impact of SARS‐CoV‐2 on ventricular volume depended on age. At the group level, older patients had greater ventricular enlargement after SARS‐CoV‐2 than younger patients (interaction between age vs. ventricular enlargement: *B* = 0.17; SE = 0.05; *p* = 0.0003). However, in the group of patients with less than 40 years of age, SARS‐CoV‐2 infection was associated with a decreased rate of enlargement of ventricles over time (because B for ventricular enlargement + B for the interaction between ventricular enlargement multiplied by age lower than 40 resulted in a negative value for the final B). This may explain the negative estimate for the impact of SARS‐CoV‐2 on ventricular volume (*B* = −7.11; SE = 2.17; *p* = 0.0013) (Table 2, Figures 2 and S1).

Only patients with more severe SARS‐CoV‐2 infection showed a reduction in MUCCA (*B* = 1.14; SE = 0.52; *p* = 0.030) (Tables 2 and S2, Figure S2).

### 3.4 Association Between Brain and Spinal Cord Volumes and Disability

The whole and regional brain volume loss, as well as changes in MUCCA, were not associated with disability. However, lower thalamic (*B* = −189.13; SE = 83.14; *p* = 0.024) and spinal cord volumes (*B* = −0.03; SE = 0.004; *p* < 0.0001) were associated with higher EDSS scores.

Patients with lower EDSS scores at the time of SARS‐CoV‐2 exhibited a greater increase in T2 lesion volume over the follow‐up period (*B* = −52.28; SE = 16.01; *p* = 0.002) (Table S3).

## 4 Discussion

This study showed a slightly increased global and regional brain volume loss following SARS‐CoV‐2 infection in clinically stable pwMS. By focusing on this cohort, we isolated the impact of COVID‐19 from typical MS‐related disease activity, offering a unique perspective on how systemic infections may influence neurodegeneration in pwMS.

Since we included only clinically stable patients without clinical disease activity or any treatment interventions during the study, the increased brain volume loss following SARS‐CoV‐2 infection may seem unexpected. However, the findings of a mildly increased whole and regional brain volume loss along with increased ventricular enlargement (observed only in older patients) after SARS‐CoV‐2, are broadly consistent with those of a previous large‐scale study in healthy controls. That study reported reduced grey matter thickness, greater damage in regions functionally connected to the olfactory cortex, a more pronounced reduction in global brain size, and greater enlargement of lateral ventricles and total cerebrospinal fluid volume in affected individuals [8]. It is also worth noting that only patients with more severe SARS‐CoV‐2 infection appeared to show a reduction in spinal cord volume, suggesting that the effects of more severe infection may extend beyond the brain to involve the entire CNS. However, this complex association requires confirmation in future studies. Our work is also consistent with research examining disability progression associated with SARS‐CoV‐2 infection, as whole‐brain atrophy has repeatedly been shown to be linked to greater physical disability progression in pwMS [2, 11, 28]. Although the recent study in 14 pwMS did not show an increased whole brain volume loss following SARS‐CoV‐2, it showed regional volume loss of the parahippocampal gyri [4], similar to a study of healthy subjects [8]. We hypothesize that the absence of associations between SARS‐CoV‐2 and global and regional volume changes in the previous study may be explained by low statistical power associated with small sample size.

We also found that brain structural changes associated with the infection were age‐dependent. For example, younger age was associated with decreased ventricle volume, while older age was linked to greater ventricular enlargement following SARS‐CoV‐2 infection. This pattern is consistent with findings from a previous study in healthy controls [8]. These results underscore the importance of preventing SARS‐CoV‐2 infection, particularly in the at‐risk elderly population, especially given the proven safety profile and efficacy of vaccines, the availability of pre‐exposure monoclonal antibody treatments against SARS‐CoV‐2, and other preventive measures [3, 19, 20, 29].

The mechanisms by which SARS‐CoV‐2 impacts the CNS likely involve secondary immunological consequences rather than direct viral invasion. These include disruption of the blood–brain barrier, tissue inflammation, hypoxia, and coagulopathy. While some studies have linked SARS‐CoV‐2 to a higher incidence of MS relapses [19], our cohort excluded patients with relapses, and no differences were observed in T1 or T2 lesion activity before and after infection. Therefore, relapse‐associated mechanisms are unlikely to explain the observed increased brain volume loss. One plausible explanation is the role of smouldering inflammation, driven by activated macrophages and microglia [30]. SARS‐CoV‐2 could amplify this process, as hyperinflammatory dysregulation of the innate immune system and pathological microgliosis are known contributors to CNS toxicity in COVID‐19. These effects may interact with pre‐existing inflammation and neurodegeneration in MS, leading to additive or nonlinear effects on brain atrophy. Mechanistically, human microglia express SARS‐CoV‐2 entry factors, such as angiotensin‐converting enzyme 2 (ACE2) and transmembrane protease serine subtype 2 (TMPRSS2), as well as activation of NOD‐like receptor pyrin domain‐containing protein (NLRP) inflammasomes, which may mediate this interaction [2]. Further research is needed to disentangle these mechanisms and determine how much of the observed atrophy can be attributed to COVID‐19, MS, or their combined effects.

Our study has several limitations. First, we did not assess the atrophy of brain regions directly associated with the olfactory system, which might be expected to suffer the most. Second, the MRI changes following SARS‐CoV‐2 were only mild. It is important to acknowledge that while a decrease in whole or regional brain volume following SARS‐CoV‐2 infection (e.g., 0.17% for whole brain volume) may be statistically significant, it may not be clinically meaningful or have significant real‐world implications. Therefore, the study would have benefited from a larger dataset and longer follow‐up periods with more imaging data to better evaluate the dynamics of change. Third, we did not have MRI sequences that allow for the direct evaluation of the presence and evolution of smouldering lesions. Fourth, our study lacks healthy controls for comparison. Fifth, although there are asymptomatic cases of SARS‐CoV‐2 infection in our cohort, it is likely that we did not capture all of them. Sixth, patients with comorbidities such as arterial hypertension or migraine were not excluded. Although the inclusion of patients with comorbidities may reflect the real‐world population of patients with MS, where such comorbidities are common and may contribute to clinical and imaging outcomes, we acknowledge that it represents a limitation of the study. We cannot exclude the possibility that patients with certain types of comorbidities may be more vulnerable to SARS‐CoV‐2‐driven brain volume loss. On the other hand, the statistical models compared brain volume loss before and/or after infection with brain volume loss during the infection in individual patients with certain comorbidities, which we expect remained relatively stable during the follow‐up. Therefore, we do not expect that the potential confounding effect of comorbidities would have had a major impact on our results at the group level. To partially address the potential confounding effects of these comorbidities, all statistical models were adjusted for age and disability (assessed using the EDSS), as these factors are at least partially correlated with a higher burden of comorbidities. Therefore, future studies should investigate the effect of different comorbidities on infection‐driven brain volume loss in patients with brain diseases, which could improve our understanding of the mechanisms of neurodegeneration. Seventh, only 10 patients in our study have undergone at least two MRI scans following SARS‐CoV‐2 infection. Therefore, we were unable to investigate whether the increased rate of volume loss persists and for how long after the infection. Lastly, it is essential to note that this study was exploratory, and *p*‐values were not corrected for multiple comparisons. Consequently, results with *p*‐values close to the alpha threshold, such as grey matter volume loss, should be interpreted cautiously. Further studies with larger cohorts and extended follow‐up periods are needed to confirm these findings.

If our results are confirmed in future research, they may have several important clinical implications. Increased brain volume loss in clinically stable patients following SARS‐CoV‐2 infection might not always reflect DMT failure but could instead be linked to a comorbidity, such as SARS‐CoV‐2. These findings could improve the interpretation of MRI findings in pwMS after infections, helping to distinguish between changes driven by the underlying disease and those associated with external factors. This highlights the importance of preventive measures, including timely vaccination and effective infection management, to mitigate risks associated with SARS‐CoV‐2 in pwMS. Future research should also explore whether other systemic or nervous system infections contribute to increased brain atrophy, further enhancing our understanding of brain volume loss trajectories in MS and informing both clinical practice and research.

## 5 Conclusion

Our study showed that SARS‐CoV‐2 in clinically stable pwMS was associated with an increased brain volume loss. In addition, patients with more severe infection appeared to show a greater reduction in spinal cord area. These findings suggest that even in the absence of clinical disease activity, SARS‐CoV‐2 may contribute to structural CNS changes in pwMS. This highlights the importance of comorbidities in monitoring and evaluating brain volume changes in pwMS and underscores the need for preventive measures against SARS‐CoV‐2 infection, particularly in the higher‐age vulnerable population. Future research should aim to confirm these results in larger cohorts or in patients with other infections. Additionally, it should investigate the underlying mechanisms driving these changes and explore the long‐term clinical implications.

## Author Contributions

**Tomas Uher:** conceptualization, methodology, statistical analysis, investigation, data curation and writing – original draft. **Dominika Stastna:** conceptualisation, investigation and writing – original draft. **Ingrid Menkyova:** investigation and data curation. **Vaclav Capek:** methodology and statistical analysis. **Jiri Lindner:** methodology and data curation. **Petra Nytrova:** conceptualization and investigation. **Jan Krasensky:** conceptualisation and methodology. **Eliza Varju:** writing – review and editing. **Miguel D'haeseleer:** conceptualization, review and editing. **Eva Kubala Havrdova:** conceptualisation and investigation. **Dana Horakova:** conceptualisation and investigation. **Manuela Vaneckova:** methodology, investigation and resources. **Niels Bergsland:** conceptualisation, methodology, software, data curation, writing – review and editing and supervision.

## Conflicts of Interest

The authors declared the following potential conflicts of interest with respect to the research, authorship, and/or publication of this article: Tomas Uher received financial support for conference travel and honoraria from Biogen, Novartis, Roche, Bristol Myers Squibb, and Merck, as well as support for research activities from Biogen and Sanofi; Dominika Stastna received financial support for conference travel and/or speaker honoraria and/or consultant fees from Novartis, Biogen, Merck, Janssen‐Cilag, and Pfizer; Petra Nytrova received speaker honoraria and consultant fees from Biogen, Novartis, Merck, and Roche, and financial support for research activities from Roche and Merck; Jan Krasensky received financial support for research activities from Biogen; Miguel D'haeseleer has received consultancy fees, educational support, and/or research grants from Merck, Teva, Biogen, Sanofi Genzyme, Novartis, Roche, Almirall, Janssen‐Cilag, and Bristol‐Myers Squibb; Eva Kubala Havrdova received speaker honoraria and consultant fees from Biogen, Merck, Novartis, Sanofi, Teva, Actelion, and Receptos, as well as support for research activities from Biogen and Merck; Dana Horakova received compensation for travel, speaker honoraria, and consultant fees from Biogen, Novartis, Merck, Bayer, and Teva, as well as support for research activities from Biogen; Manuela Vaneckova received speaker honoraria and consultant fees from Biogen, Novartis, Roche, Sanofi, and Teva, as well as support for research activities from Biogen; other authors declare no conflicts of interest.

## Supporting information


**Table S1.** Structure of the statistical model.
**Table S2.** Impact of the SARS‐Cov‐2 on MRI measures in clinically stable patients with multiple sclerosis. Statistical models were adjusted for MS phenotype, but not for disability status assessed by EDSS.
**Table S3.** Association between evolution of MRI measures and disability status assessed by EDSS.

**Figure S1.** Association between changes in ventricles volume before and after SARS‐CoV‐2 and age at infection.

**Figure S2.** Association between changes in mean upper cervical cord area (MUCCA) before and after SARS‐CoV‐2 and infection severity (0: No infection; 1: Asymptomatic; 2: Symptomatic without suspected pneumonia; 3: Suspected pneumonia defined by both dry cough and shortness of breath; 4: Radiologically confirmed pneumonia; 5: Need of supplemental oxygen; 6: Need of non‐invasive ventilation or high‐flow oxygen therapy).

## Data Availability

Essential data relevant to the study are included in the article. The data underlying this article are stored in a protected server environment managed by the ReMuS registry and cannot be shared publicly due to data protection regulations. Access to the data is granted to authorised researchers after they have made a request to the ReMuS Scientific Board and the Registry Management Board, provided that their request complies with ethical standards and data protection laws.
