# Supplementary material for: SARS‐CoV‐2 Is Linked to Brain Volume Loss in Multiple Sclerosis
Source: Ann Clin Transl Neurol. 2025 May 29;12(8):1548–55. doi: 10.1002/acn3.70091 (PMC12343308; doi:10.1002/acn3.70091)
Supplement: Supplementary file 1 — Table S1. Structure of the statistical model. Table S2. Impact of the SARS‐Cov‐2 on MRI measures in clinically stable patients with multiple sclerosis. Statistical models were adjusted for MS phenotype, but not for disability status assessed by EDSS. Table S3. Association between evolution of MRI measures and disability status assessed by EDSS. [file ACN3-12-1548-s002.docx]

**Supplementary Table 1**. Structure of the statistical model.

| Dependent variable | Independent variable | | |
| --- | --- | --- | --- |
| Annualised MRI measure relative or absulute change (Continuous) | SARS-CoV-2 | Categorical | 0: No infection; 1: The first MRI scans after infection |
|  | Time | Continuous | Number of days between infection and MRI scan |
|  | Sex | Categorical | Female/Male |
|  | Age at the SARS-CoV-2 | Continuous | -- |
|  | EDSS at the SARS-CoV-2 | Continuous | Supplementary Table 2 shows results of models adjusted for categorical variable MS phenotype (Relapsing-remitting MS/Secondary progressive MS), but not for disability status assessed by EDSS. |
|  | DMT status at the SARS-CoV-2 infection | Categorical | From 1 to 4 (1: Untreated; 2: low efficacy [dimethyl-fumarate, glatiramer acetate, interferon β, teriflunomide]; 3: moderately-high efficacy [cladribine and fingolimod]; and 4: high-efficacy monoclonal antibodies DMTs [anti-CD20, alemtuzumab, and natalizumab]) |
|  | Severity of the SARS-CoV-2 infection | Continuous | From 0 to 6 (0: No infection; 1: Asymptomatic; 2: Symptomatic without suspected pneumonia; 3: Suspected pneumonia defined by both dry cough and shortness of breath; 4: Radiologically confirmed pneumonia (chest X-ray or CT scan); 5: Need of supplemental oxygen; 6: Need of non-invasive ventilation or high-flow oxygen therapy |
|  | Anti-SARS-CoV-2 treatment | Categorical | Yes/No |

Detail of the statistical model:

MRI measure ~ SARS-CoV-2 * (Time + Sex + Age + EDSS + DMT status) + Severity of the SARS-Cov-2 + Anti-SARS-Cov-2 treatment + (1 + MRI measure | Patient identifier)

*Interaction term

1 | Patient identifier = random intercept

MRI measure | Patient identifier = random slope

**Supplementary Table 2.** Impact of the SARS-Cov-2 on MRI measures in clinically stable patients with multiple sclerosis. Statistical models were adjusted for MS phenotype, but not for disability status assessed by EDSS.

| MRI measure | | Statistical analysis | | |
| --- | --- | --- | --- | --- |
|  |  | B | SE | p-value |
| Lesions  (annualised) | T1 lesion number change | 0.02 | 0.39 | 0.97 |
|  | T1 lesion volume change (mm^3^) | -100.92 | 51.46 | 0.051 |
|  | T2 lesion number change | -0.61 | 2.55 | 0.81 |
|  | T2 lesion volume change (mm^3^) | -17.07 | 57.95 | 0.77 |
| Brain volumes (annualised) | Ventricles volume change (%) | -7.11 | 2.17 | 0.0013 |
|  | Whole brain volume change (%) | -0.17 | 0.08 | 0.028 |
|  | Gray matter volume change (%) | -0.25 | 0.12 | 0.040 |
|  | Cortical volume change (%) | -0.32 | 0.13 | 0.014 |
|  | White matter volume change (%) | 0.44 | 0.70 | 0.53 |
|  | Thalamic volume change (%) | 0.38 | 1.48 | 0.80 |
| Spinal cord  (annualised) | MUCCA change (%) | 1.14 | 0.52 | 0.030 |

Legend: B = unstandardised beta coefficient from the linear mixed model; MUCCA = Mean Upper Cervical Cord Area; SE = standard error

The structure of the statistical models is shown in Supplementary Table 1.

| MRI measure | | Statistical analysis | | |
| --- | --- | --- | --- | --- |
|  |  | B | SE | p-value |
| Lesions  (annualised) | T1 lesion number change | -0.02 | 0.08 | 0.84 |
|  | T1 lesion volume change (mm^3^) | -9.32 | 7.44 | 0.21 |
|  | T2 lesion number change | 0.04 | 0.18 | 0.81 |
|  | T2 lesion volume change (mm^3^) | -52.28 | 16.01 | 0.002 |
| Brain volumes (annualised) | Ventricles volume change (%) | -0.02 | 0.33 | 0.96 |
|  | Whole brain volume change (%) | -0.001 | 0.04 | 0.98 |
|  | Gray matter volume change (%) | 0.01 | 0.08 | 0.87 |
|  | Cortical volume change (%) | 0.02 | 0.08 | 0.77 |
|  | White matter volume change (%) | -0.007 | 0.05 | 0.90 |
|  | Thalamic volume change (%) | 0.09 | 0.12 | 0.45 |
| Spinal cord  (annualised) | MUCCA change (%) | -0.09 | 0.16 | 0.58 |

**Supplementary Table 3.** Association between evolution of MRI measures and disability status assessed by EDSS.

Legend: B = unstandardised beta coefficient from the linear mixed model; MUCCA = Mean Upper Cervical Cord Area; SE = standard error

The structure of the statistical models is shown in Supplementary Table 1.
