# Supplementary figures and images for: SARS‐CoV‐2 Is Linked to Brain Volume Loss in Multiple Sclerosis
Source: Ann Clin Transl Neurol. 2025 May 29;12(8):1548–55. doi: 10.1002/acn3.70091 (PMC12343308; doi:10.1002/acn3.70091)

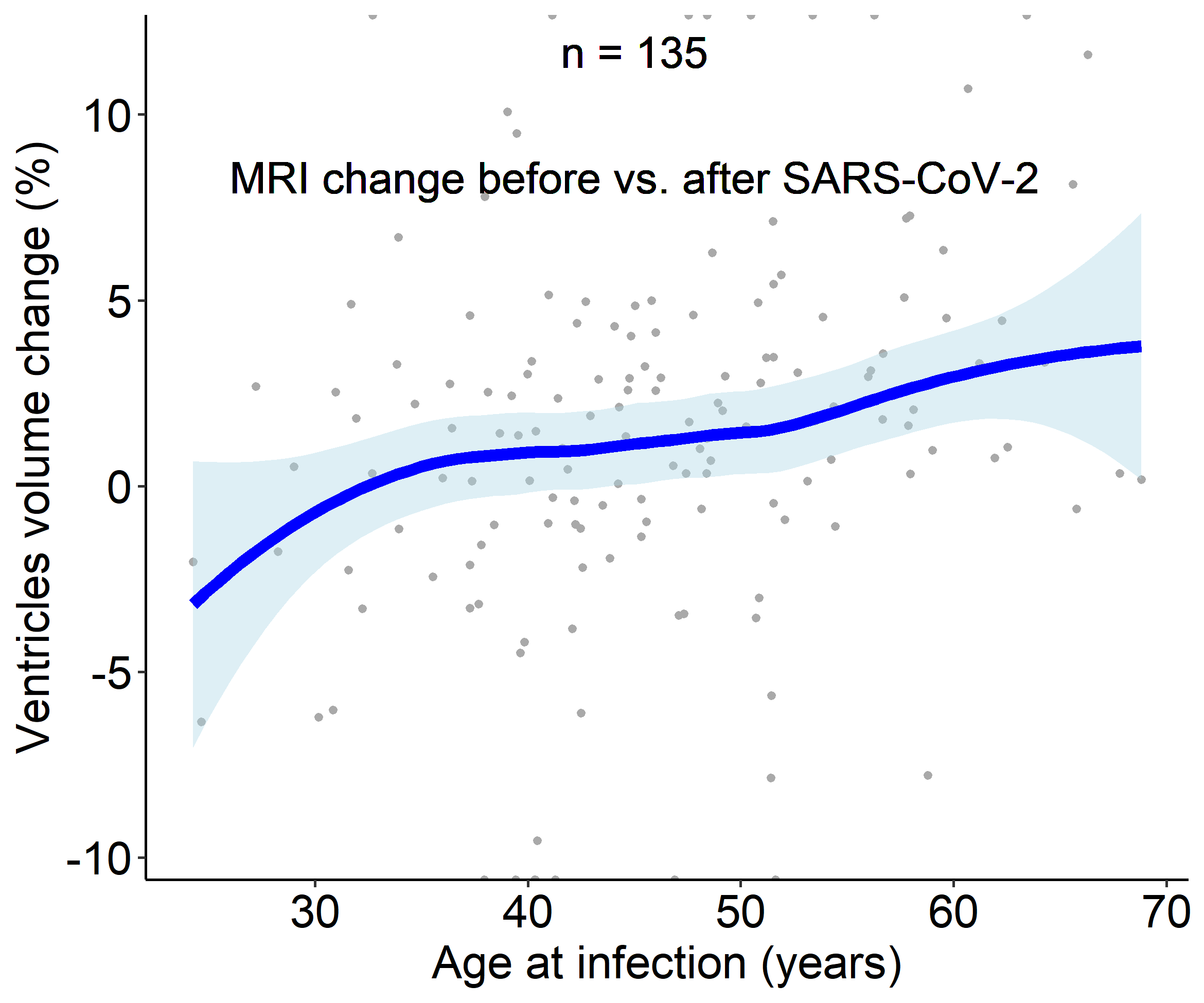

Supplement: Supplementary file 2 — Figure S1. Association between changes in ventricles volume before and after SARS‐CoV‐2 and age at infection. [file ACN3-12-1548-s003.tiff]

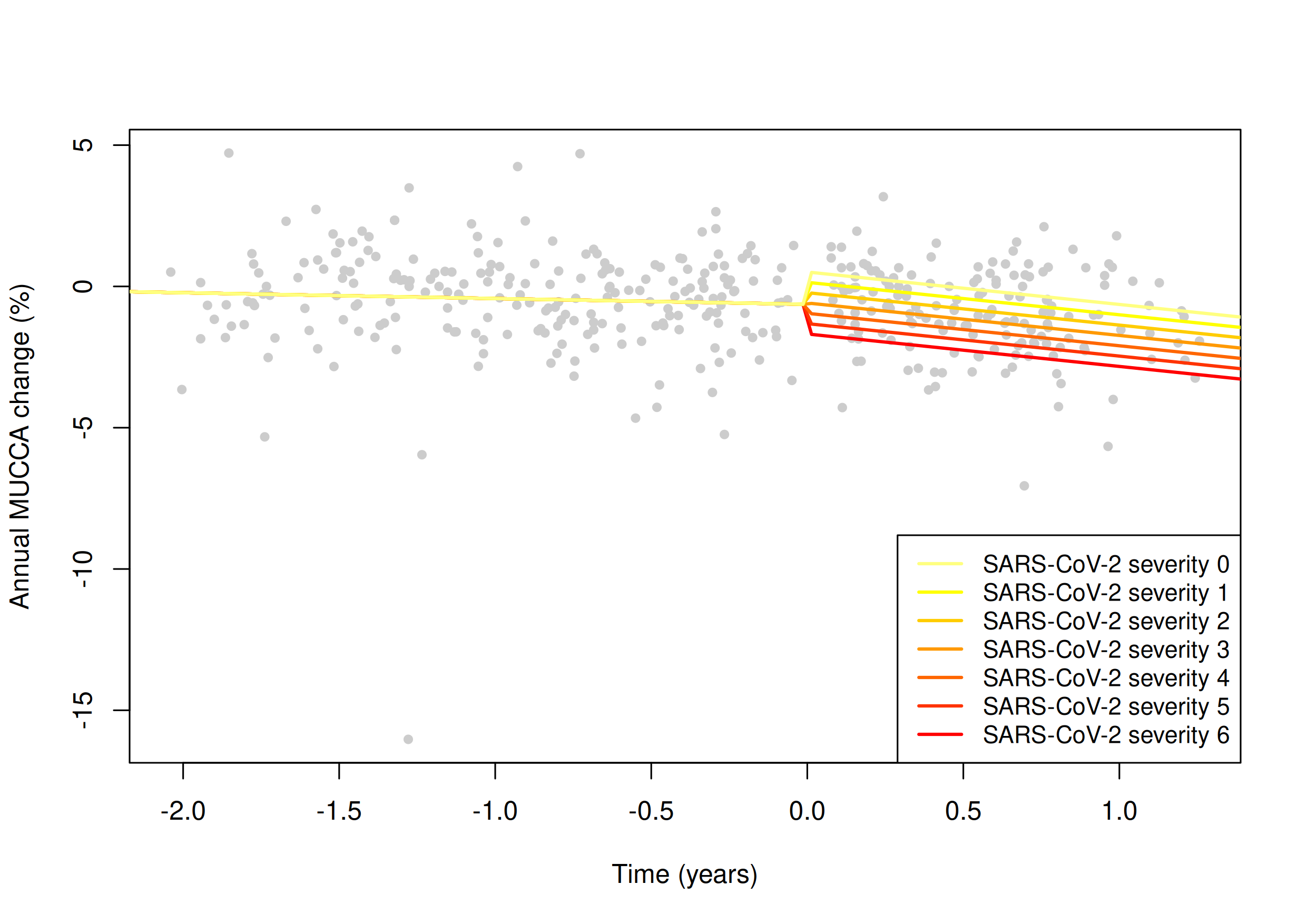

Supplement: Supplementary file 3 — Figure S2. Association between changes in mean upper cervical cord area (MUCCA) before and after SARS‐CoV‐2 and infection severity (0: No infection; 1: Asymptomatic; 2: Symptomatic without suspected pneumonia; 3: Suspected pneumonia defined by both dry cough and shortness of breath; 4: Radiologically confirmed pneumonia; 5: Need of supplemental oxygen; 6: Need of non‐invasive ventilation or high‐flow oxygen therapy). [file ACN3-12-1548-s001.tiff]
